# Supplementary figures and images for: Mutagenesis and structural modeling implicate RME-8 IWN domains as conformational control points
Source: PLoS Genet. 2022 Oct 24;18(10):e1010296. doi: 10.1371/journal.pgen.1010296 (PMC9642905; doi:10.1371/journal.pgen.1010296)

A

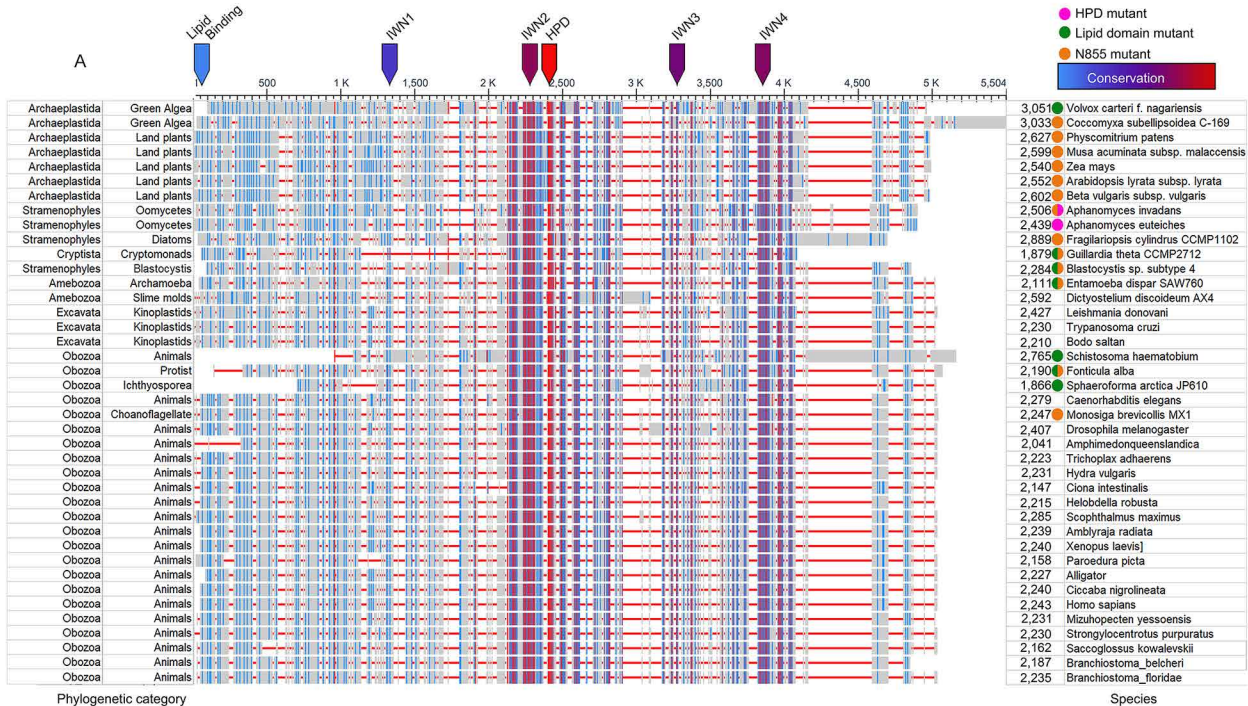

B

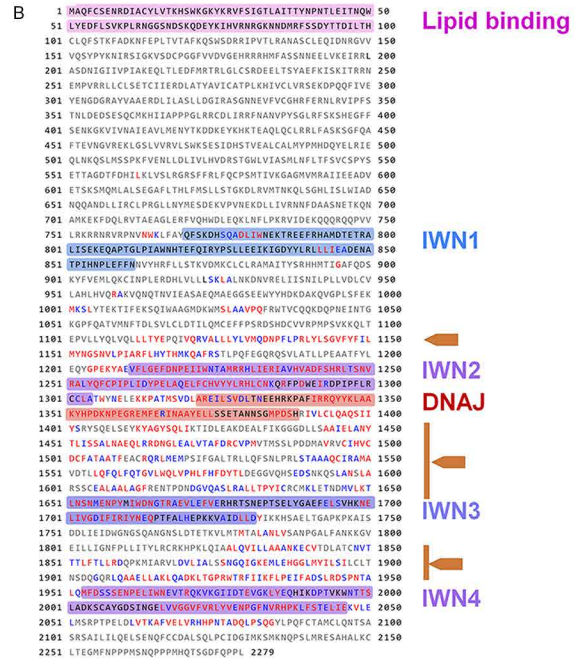

Supplement: S1 Fig — (A) Expansion of Fig 1C with more organisms included. (B) Annotated display of C. elegans RME-8 sequence from the multiple sequence alignment in Fig 1B. The defined lipid binding domain from [6] is indicated in pink. IWN repeats defined by [2] purple. The DNAJ domain is highlighted in orange. Light brown arrows indicate regions of high conservation that lie outside of established functional domains. Individual amino acids are colored according to conservation across Eukarya with grey indicating no conservation, blue indicating moderate conservation and red indicating high conservation. (PDF) [file pgen.1010296.s001.pdf]

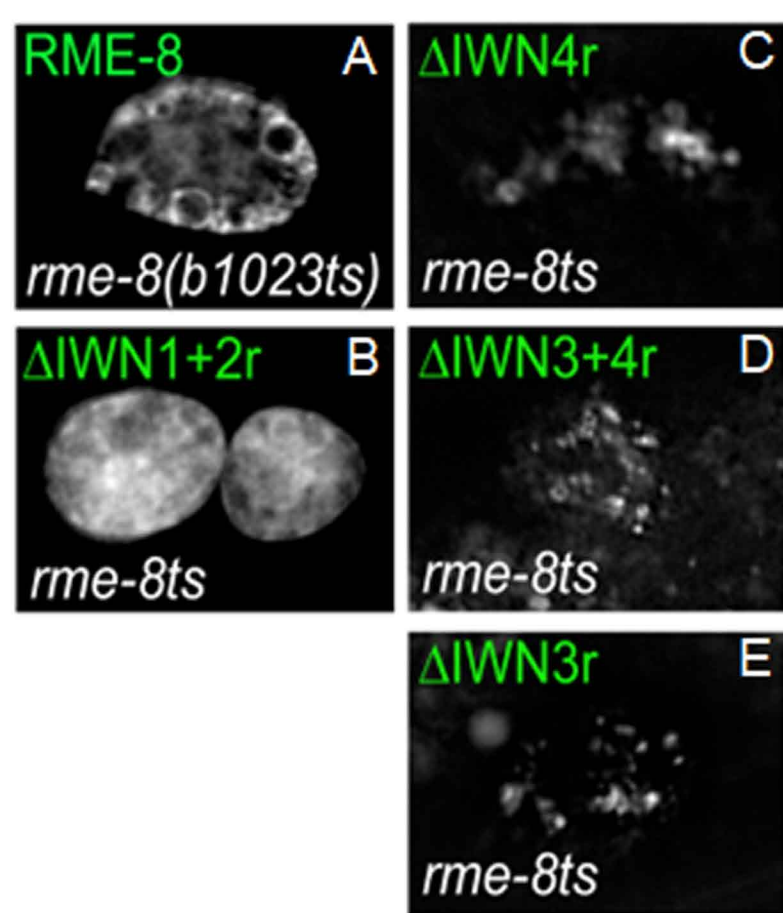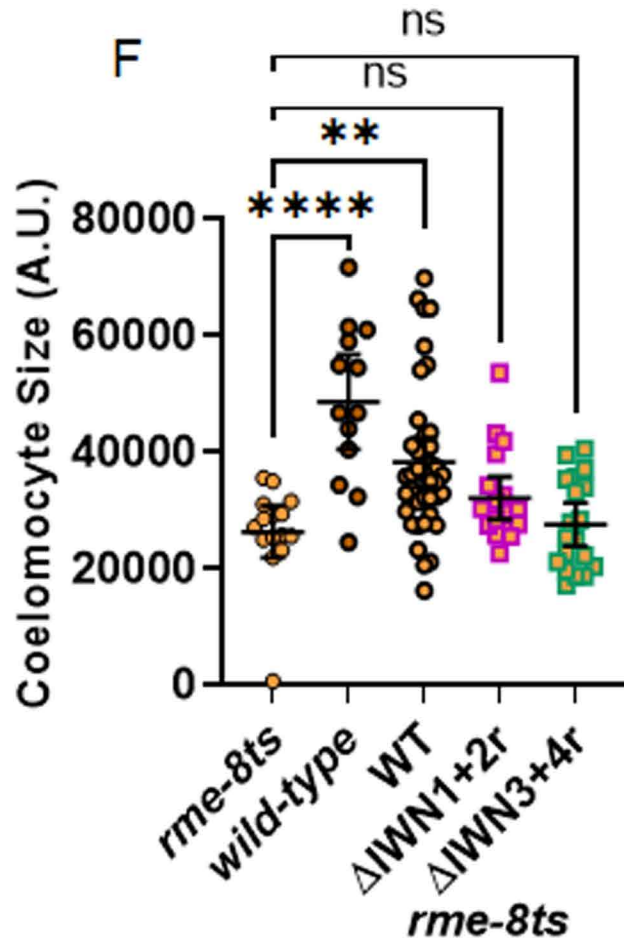

Supplement: S2 Fig — - (A-E) Micrograph of pCUP-4::GFP::RME-8 full length, N-terminal and C-terminal truncations illustrated in Fig 2J expressed in the rme8- ts mutant background at the restrictive temperature. Scale bars are 5 microns in whole coelomocyte images. (F) Quantification of coelomocyte size in rme-8ts mutant animals expressing ectopic RME-8+, N-terminal, or C-terminal truncations. (P). (PDF) [file pgen.1010296.s002.pdf]

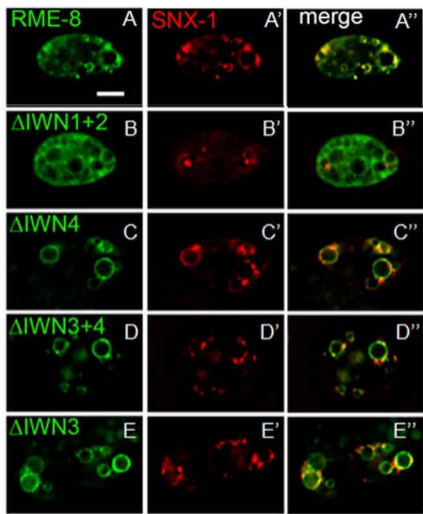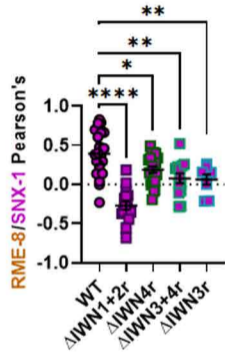

F

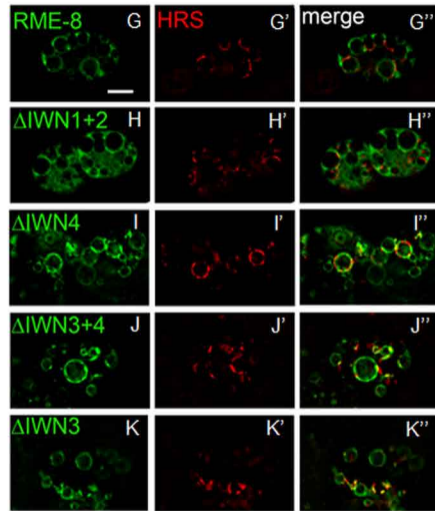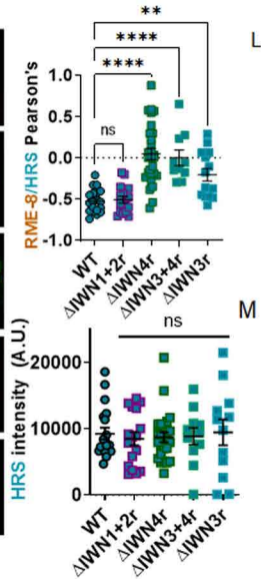

Supplement: S3 Fig — (A-E") Micrograph of pCUP-4::GFP::RME-8 wild-type, N-terminal, and C-terminal truncations co-expressed with tagRFP::SNX-1 in coelomocytes of wild-type animals. (F) Quantification of colocalization of pCUP-4::GFP::RME-8 wild-type and truncations with tagRFP::SNX-1 in coelomocytes of wild-type animals. (G-K") Micrograph of pCUP-4::GFP::RME-8 wild-type, N-terminal, and C-terminal truncations co-expressed with tagRFP::HRS(HGRS-1) in coelomocytes of wild-type animals. (L) Quantification of colocalization of GFP::RME-8 wild-type and truncations with tagRFP::HRS(HGRS-1)in wild-type animals. (M) Quantification of HGRS-1 intensity in animals expressing pCUP-4::GFP::RME-8 wild-type and truncations. (PDF) [file pgen.1010296.s003.pdf]

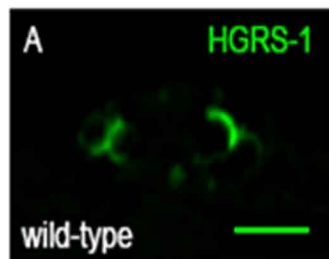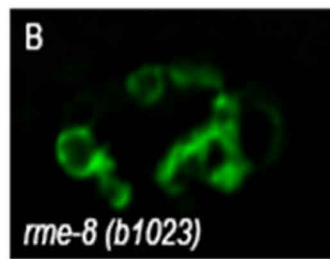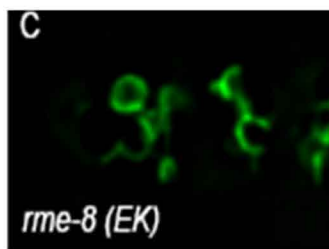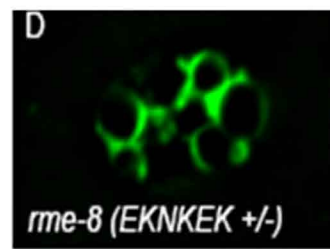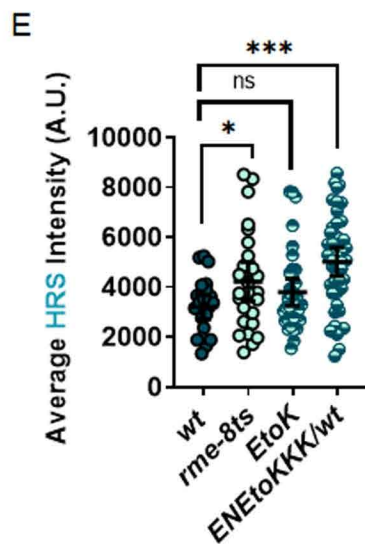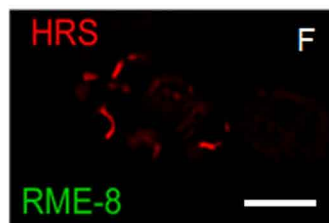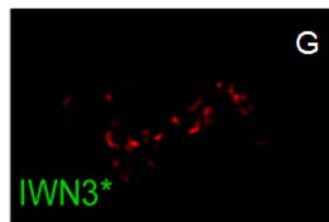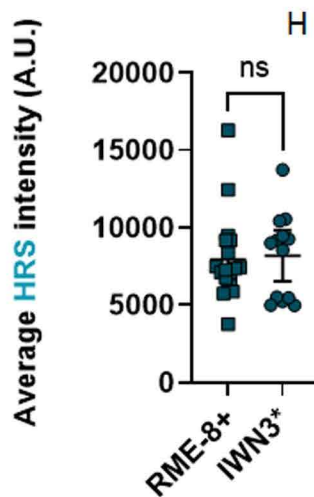

Supplement: S5 Fig — (A-D) Micrograph of pSNX-1::Citrine::HRS/HGRS-1 expressed in coelomocytes of wild-type (A) rme-8ts(b1023) animals (B), CRISPR generated rme-8 E1962K animals (C), and heterozygous CRISPR generated rme-8 (E1962K/N1966K/E1967K) animals(D). (E) Quantification HRS/HGRS-1 average intensity animals represented in A-D. (F-G)) Micrograph of tagRFP::HRS(HGRS-1) in wild-type animals expressing pCUP-4::GFP::RME-8+ or D1657K mutation in IWN3 (IWN3*). Quantification HRS/HGRS-1 average intensity animals represented in F-G. Each data point is an individual worm, error bars indicate Mean with 95% CI, student t-test statistical analysis done in Prism with p < .5 = *, p < .01 = **, p < .001 = ***. Scale bars are 5 microns in whole coelomocyte images. (PDF) [file pgen.1010296.s005.pdf]
