# Supplementary material for: Mutagenesis and structural modeling implicate RME-8 IWN domains as conformational control points
Source: PLoS Genet. 2022 Oct 24;18(10):e1010296. doi: 10.1371/journal.pgen.1010296 (PMC9642905; doi:10.1371/journal.pgen.1010296)
Supplement: S4 Fig — (A) Empty vector or SNX-1 (aa221-472) expressed in pDEST22 tested for interaction with of the RME-8 C-terminus with DNAJ domain. The RME-8 fragments were expressed in pDEST32 using JDY27 containing URA3, ADE2, and HIS3 reporter genes. 5ul of suspended yeast at 1, and 0.1 OD’s were spotted on SC-LEU-TRP growth, or assay plates SC-LEU-TRP -URA. Lysines and Arginines of helix II of the DNAJ domain were targeted for doped oligo mutagenesis and selected for increased interaction with SNX-1. The aa1322-2279 fragment with lysines at position 1347 and 1356 substituted with glutamic acid was sufficient to show growth on assay media when combined with SNX-1 BAR domain, singly these mutations were not able to grow on the assay media. Arginines 1342 and 1342 were also substituted with glutamic acid and failed to grow well on the assay media. (B) Empty vector or SNX-1 (aa221-472) expressed in pDEST22 tested for interaction with of the RME-8 C-terminus with our without its DNAJ domain, and mutants isolated in the screen. The RME-8 fragments were expressed in pDEST32 using JDY27 containing URA3, ADE2, and HIS3 reporter genes. 5ul of suspended yeast at 1, and 0.1 OD’s were spotted on SC-LEU-TRP growth, or assay plates with increasing stringency; SC-LEU-TRP-HIS, SC-LEU-TRP-HIS+25mM 3AT, and SC-LEU-TRP-URA. A schematic representation of the RME-8 fragments tested are illustrated to the left of the yeast two hybrid assay. (B) A schematic of the Fragment mutated represented to the left of the assay. 5ul of suspended yeast at 1, and 0.1 OD’s were spotted on SC-LEU-TRP growth or SC-LEU-TRP-URA assay media. (PDF) [file pgen.1010296.s004.pdf]

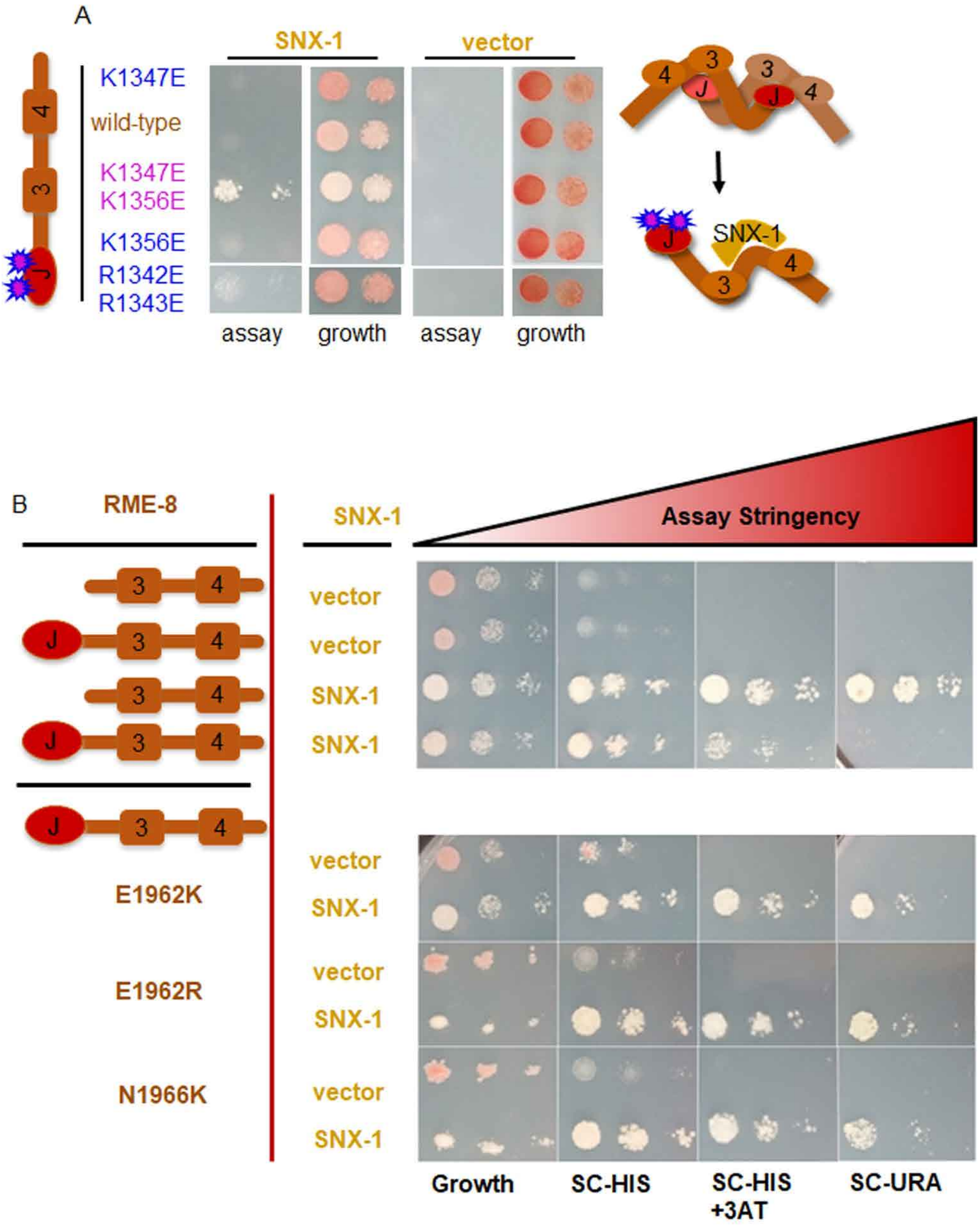
A
SNX-1
vector
K1347E
wild-type
K1347E
K1356E
K1356E
R1342E
R1343E
4
3
J
assay
growth
assay
growth
SNX-1
B
RME-8
SNX-1
Assay Stringency
vector
vector
SNX-1
SNX-1
E1962K
vector
SNX-1
E1962R
vector
SNX-1
N1966K
vector
SNX-1
Growth
SC-HIS
SC-HIS
+3AT
SC-URA
