# Supplementary material for: Mutagenesis and structural modeling implicate RME-8 IWN domains as conformational control points
Source: PLoS Genet. 2022 Oct 24;18(10):e1010296. doi: 10.1371/journal.pgen.1010296 (PMC9642905; doi:10.1371/journal.pgen.1010296)
Supplement: S1 Tablestylefix — (PDF) [file pgen.1010296.s006.pdf]

Strain	Genotype	Reference	Figure
RT4119	pwSi237[pSNX-1 GFP RME-8(minigene) in pcfj1662]	This study	2
RT4124	pwSi292 [pSNX-1 GFP RME-8(minigene Δ1661-2279)in pcfj1662]	This study	2
RT2788	pwIs1054[psnx-1::tagRFP::RAB-5genomic::unc54 3"UTR in pcfj150]-cBUnc119;	This study	2
RT2880	pwIs1117[psnx-1::tagRFP::RAB-7(genomic)::unc54 3"UTR in pcfj150]-cBUnc119;	This study	2
RT4282	pwIs984[psnx-1::tagRFP::snx-1genomic]-cBUnc119; pwsi237 [pSNX-1 GFP RME-8(minigene) in pcfj1662];	This study	3
RT4284	pwIs984[psnx-1::tagRFP::snx-1genomic]-cBUnc119; pwSi290 [pSNX-1 GFP RME-8(minigene Δ101-1387)] in pcfj1662	This study	3
RT4286	pwIs984[psnx-1::tagRFP::snx-1genomic]-cBUnc119; pwSi292 [pSNX-1 GFP RME-8(minigene Δ1661-2279)in pcfj1662]	This study	3
RT4150	pwSi290 [pSNX-1 GFP RME-8(minigene Δ101-1387)] in pcfj1662; pwIS1038[psnx-1::tagRFP::HGRS-1-unc54-3'UTR in pCFJ150]; rme-8(b1023ts)	This study	3
RT4151	pwSi292 [pSNX-1 GFP RME-8(minigene Δ1661-2279)in pcfj1662]; pwIS1038[psnx-1::tagRFP::HGRS-1-unc54-3'UTR in pCFJ150]; rme-8(b1023ts)	This study	3
RT4152	pwIS1038[psnx-1::tagRFP::HGRS-1-unc54-3'UTR in pCFJ150]; rme-8(b1023ts)	This study	3
RT4130	pwSi290 [pSNX-1 GFP RME-8(minigene Δ101-1387)] in pcfj1662; pwIS1038[psnx-1::tagRFP::HGRS-1-unc54-3'UTR in pCFJ150];	This study	3
RT4123	pwSi292 [pSNX-1 GFP RME-8(minigene Δ1661-2279)in pcfj1662] ; pwIS1038[psnx-1::tagRFP::HGRS-1-unc54-3'UTR in pCFJ150];	This study	3
RT4530	pwSi492[pSNX-1 GFP RME-8(minigene IWN4*) in pcfj1662]; pwIS1038[psnx-1::tagRFP::HGRS-1-unc54-3'UTR in pCFJ150];rme-8(b1023ts)	This study	5
RT4136	pwSi257[pSNX-1 GFP RME-8(minigene IWN3*) in pcfj1662]; pwIS1038[psnx-1::tagRFP::HGRS-1-unc54-3'UTR in pCFJ150];rme-8(b1023ts)	This study	5
RT4283	pwIs984[psnx-1::tagRFP::snx-1genomic]-cBUnc119; pwSi257 [pSNX-1 GFP RME-8(minigene IWN3*) in pcfj1662]	This study	6
RT3692	pwSi257[pSNX-1 GFP RME-8(minigene IWN3*) in pcfj1662]; pwIs1053 [psnx-1tagRFP::RME-8(minigene)::unc54 3'UTR]	This study	6
RT3680	pwSi237[pSNX-1 GFP RME-8(minigene) in pcfj1662]; pwIs1053 [psnx-1tagRFP::RME-8(minigene)::unc54 3'UTR]	This study	6

DH1336	bIs34[rme-8::GFP, rol6(d)]	Zhang et al. 2001	6
RT1233	bIs34[rme-8::GFP, rol6(d)]; tm847 (snx-1)	Shi et al 2009	6
RT225	pwIs94[gfp::snx-1 gen];(unc-119(ed3)	Shi et al 2009	7
RT4155	pwIs984[psnx-1::tagRFP::snx-1genomic]-cBUnc119; rme-8(b1023ts)	This study	7
RT4276	pwIs984[psnx-1::tagRFP::snx-1genomic]-cBUnc119; pwsi237[pSNX-1 GFP RME-8(minigene) in pcfj1662];; rme-8(b1023ts)	This study	7
RT4277	pwIs984[psnx-1::tagRFP::snx-1genomic]-cBUnc119; pwSi257[pSNX-1 GFP RME-8(minigene IWN3*) in pcfj1662]; rme-8(b1023ts)	This study	7
RT4278	pwIs984[psnx-1::tagRFP::snx-1genomic]-cBUnc119; pwSi290 [pSNX-1 GFP RME-8(minigene Δ101-1387)] in pcfj1662; rme-8(b1023ts)	This study	7
RT4280	pwIs984[psnx-1::tagRFP::snx-1genomic]-cBUnc119; pwSi292 [pSNX-1 GFP RME-8(minigene Δ1661-2279)in pcfj1662]; rme-8(b1023ts)	This study	7
RT1307	pwIs94[gfp::snx-1 gen];(unc-119(ed3); rme-8(b1023ts)	Shi et al 2009	7
RT4122	pwSi293 [pSNX-1 GFP RME-8(minigene Δ1388-1950)in pcfj1662]; rme-8(b1023ts)	This study	sup
RT4285	pwIs984[psnx-1::tagRFP::snx-1genomic]-cBUnc119; pwSi291 [pSNX-1 GFP RME-8(minigene Δ1951-2279)in pcfj1662]	This study	sup
RT4126	pwSi291[pSNX-1 GFP RME-8(minigene Δ1951-2279)in pcfj1662]; rme-8(b1023ts)	This study	sup
RT4287	pwIs984[psnx-1::tagRFP::snx-1genomic]-cBUnc119; pwSi293 [pSNX-1 GFP RME-8(minigene Δ1388-1950)in pcfj1662]	This study	sup
RT4153	pwSi291 [pSNX-1 GFP RME-8(minigene Δ1951-2279)in pcfj1662]; pwIS1038[psnx-1::tagRFP::HGRS-1-unc54-3'UTR in pCFJ150]; rme-8(b1023ts)	This study	sup
RT4154	pwSi293 [pSNX-1 GFP RME-8(minigene Δ1388-1950)in pcfj1662; pwIS1038[psnx-1::tagRFP::HGRS-1-unc54-3'UTR in pCFJ150]; rme-8(b1023ts)	This study	sup
RT4279	pwIs984[psnx-1::tagRFP::snx-1genomic]-cBUnc119; pwSi291 [pSNX-1 GFP RME-8(minigene Δ1951-2279)in pcfj1662]; rme-8(b1023ts)	This study	sup
RT4281	pwIs984[psnx-1::tagRFP::snx-1genomic]-cBUnc119; pwSi293 [pSNX-1 GFP RME-8(minigene Δ1388-1950)in pcfj1662; rme-8(b1023ts)	This study	sup
RT4125	pwSi291 [pSNX-1 GFP RME-8(minigene Δ1951-2279)in pcfj1662]	This study	sup

RT4127	pwSi291 [pSNX-1 GFP RME-8(minigene Δ1951-2279)in pcfj1662]; pwIS1038[psnx-1::tagRFP::HGRS-1-unc54-3'UTR in pCFJ150];	This study	sup
RT4120	pwSi293 [pSNX-1 GFP RME-8(minigene Δ1388-1950)in pcfj1662]	This study	sup
RT4121	pwSi293 [pSNX-1 GFP RME-8(minigene Δ1388-1950)in pcfj1662]; pwIS1038[psnx-1::tagRFP::HGRS-1-unc54-3'UTR in pCFJ150];	This study	sup
