# Supplementary material for: Mutagenesis and structural modeling implicate RME-8 IWN domains as conformational control points
Source: PLoS Genet. 2022 Oct 24;18(10):e1010296. doi: 10.1371/journal.pgen.1010296 (PMC9642905; doi:10.1371/journal.pgen.1010296)
Supplement: Attachment — Submitted filename: plos genetics rebuttal 09022022.docx [file pgen.1010296.s024.docx]

Dear Dr. Sato and Reviewers

Thank you all for your all your time and effort in reviewing our work. We especially appreciate all the positive feedback. It is both humbling and encouraging to receive such feedback.

We hope that you all will find that we have carefully considered each reviewer’s comments and criticisms. We have edited the manuscript for clarity and for conceptual ideas brought up by reviewers. We have also added data and clarified some of the figures to address reviewer concerns. We feel the manuscript has been much improved because of the feedback. Please find below our answers to each reviewer’s questions/and or suggestions for revision (written in blue).

Reviewer #1: Norris et al. present the first molecular hints to how the large RME-8 protein regulates endosomal microdomain formation. Using an evolutionary analysis, they identify new conserved domains in the protein and lay the groundwork for years if not decades of new discoveries. They delve deeper into the function of the IWN domains, revealing key roles of the third and fourth IWN domains with deletion and point mutants as molecular hinges regulating physical interactions with the DnaJ domain and SNX-1. Finally, they propose a mechanistic model of RME-8 regulation, informed by their structure-function data as well as AlphaFold structure predictions. This study represents an important step forward for understanding microdomains and is likely to be highly impactful given the breadth of information that it reveals.

Response to reviewer #1

We are very pleased that the reviewer thinks this work is very impactful and lays the groundwork for work on RME-8 for years to come. These kind words are very encouraging.

Major suggestions

All searches for RME-8 homologs in Fig. 1 were performed using RME-8 from a single species. Would more divergent homologs be identified by searching with more closely related species? For example, if you search fungal proteomes with a sponge or choanoflagellate RME-8 homolog, do you still find nothing passing the criteria? Similarly, searching gymnosperm proteomes with an angiosperm RME-8 or alveolate proteomes with an amoeba RME-8 would strengthen the claim that RME-8 is absent in these lineages.

This is an interesting question, and something we did initially. What makes RME-8 so unique is that it is the largest DNAJ domain containing protein by far. By using the cutoff of 40% query coverage in our BLAST we eliminated many hits that had quite low e values, given that DNAJ domains are conserved so broadly.

Specifically, using either choanoflagellate to query fungi or amoeba to query alveolates we do not find any genes that meet these criteria. For the gymnosperm group, however, our new BLAST does pick up a lone RME-8 homolog in Chinese Yew. Importantly, this is the only gymnosperm we detected, it is a newly deposited sequence, and it is detected regardless of whether I use a C. elegans RME-8 or Angiosperm RME-8

I have altered the text to add that these homolog searches are a snapshot, as evolutionary trees change the more sequence information we get and our understand of evolution changes. Line # 114-117

In Figure 2G&J, the constructs are labeled ∆N-term, but Fig. 2E, H & I are labeled ∆IWN1+2. Considering that these large deletions also remove the newly identified conserved domains in addition to the IWN domains, it would be helpful to include the new yellow domains in the diagrams in Fig. 2G (or better using a to-scale block cartoon, the cartoons from Fig. 9, or the AlphaFold models) and to be consistent in the naming throughout the figure. Similarly, the discussion of the C-terminal deletion should be more cautious about assigning the microdomain function to IWN3/4 in lines 156-159, given the other domains in the deletion.

This is a good point. I resist naming it ∆N-term because it retains the lipid binding domain as defined by [29]. Moreover, when we did these experiments alphafold did not exist, and we had no idea that the lipid domain would potentially extend further, nor did we have any idea there were distinct alpha solenoid regions in the C-terminus of RME-8.

To address the issue, I have renamed the deletions adding “r” for regions and made the deleted regions clearer in the figures. Also, I have added the newly identified conserved domains (formerly yellow) to the cartoons and made them to scale in both figure 1 and figure 2. Finally, in figure 2 I have included data that was originally in the supplement to justify using the ∆IWN3r and/or ∆IWN4r terminology.

Line 149-151 - Figure 3D does not quantify SNX-1 spread, because it is quantifying colocalization with RME-8. That will change based on the observed changes to RME-8 localization from Fig. 2. It would be more informative to perform an analysis similar to Fig. 2I for SNX-1 spread. The similar criticism applies for using Fig. 3H to measure HRS spread in the previous sentence, although that is more carefully worded, as well as a separate analysis of RME-8 spread and HRS spread in the IWN3* and IWN4* mutants in Fig. 5 and RME-8 spread and SNX-1 spread in Fig. 6.

This is a fair point, and I reworded the sentences to better reflect our measurement goals. See lines 202-204. Mechanistically, I would have sought to measure SNX-1 spread if the RME-8 truncations had increased SNX-1 colocalization, indicating a likelihood that they still bind to SNX-1 *in vivo*. Given that the colocalization decreased, despite a large increased spread in RME-8 suggests that these nonfunctional truncations do not bind SNX-1 *in vivo*. A key aspect of the model is that both the nonfunctional C-terminal truncations *and* the hyperactive IWN3* allele both lose colocalization with SNX-1 but have very different effects on HRS accumulation and RME-8 microdomain positioning.

Fig. 4A - Has the IWN4 been tested to bind SNX-1 on its own (or a construct like aa ~1670-2279 in Y2H)? It’s strange to refer to the 1388-2279 region as the minimal domain in line 215 without evidence that removing the IWN3 sequence is detrimental to SNX-1 binding. Given the results of the D1657K experiment in Fig. 4B, the SNX-1 binding region may map into the sequence C-terminal to IWN3, such as the newly identified yellow domain. The DnaJ domain and SNX-1 seem likely to bind slightly different sites in the C-terminal region, but still sterically hinder the other.

My apologies for lack of clarity. The experiments defining the minimal SNX-1 binding region were done in a previous publication (Shi et al, 2009)[9] from our lab. 1388-2279 is the optimal region (this study), while 1388-1950 is the minimal (this study and Shi et al 2009). 1388-1731 (contains IWN3) was unable to interact with SNX-1 (Shi et al 2009).

A great test of the author’s uncoating model would be to make complementary charge mutations in the DnaJ and IWM3 domains to restore the intramolecular interaction and allow SNX-1 binding while disrupting the Hsc70 interaction. Do AlphaFold multimer predictions using separate DnaJ and IWM3-4 domains allow the authors to guess whether D1657 is likely to interact with K1347 or K1356? Then a D1657K K13**D double mutant could be informative in their Y2H and in vivo assays.

Thanks, we have investigated this to some extent but did not include it since negative results were hard to interpret. We have tested AlphaFold multimer and it is not able to detect a meaningful interaction between the DNAJ domain and the RME-8 C-terminus; elucidation likely requires experimentally derived structural information. We tested some combinations in yeast two hybrid for restored interaction but did not obtain positive results. We feel that testing all combinations is beyond the scope of the current study. In vivo testing may be hard to interpret since disrupting the DNAJ domain obliterates both the activity and the normal microdomain of RME-8, causing the endosomes to clump and lose their circular profiles (Norris, 2017).

What are the effects of IWN4* on SNX-1 localization (i.e. Fig. 6-7)?

IWN4* did not affect SNX-1 localization. I have added IWN4* data to figure 4.

It’s not clear how the authors draw a conclusion on RME-8 acting independently of SNX-1 in line 271-272 by examining a mutant that increases SNX-1 binding.

IWN3* reduced DNAJ-binding, but unlike IWN4*, did not measurably increase SNX-1 binding. Importantly, IWN3* is hyperactive for HRS uncoating despite a stark decrease in SNX-1 colocalization, arguing that RME-8 IWN3* is active even when not SNX-1 bound. Combined with other observations, such as the effects of loss of RME-8 on SNX-1 dynamics, we propose that wild-type RME-8 depends upon SNX-1 for activation/de-homo-oligomerization, but may remove SNX-1 before uncoating other substrates such as HRS.

What is the effect of a snx-1 mutant on IWN3* localization? Would it be similar to wild type RME-8?
We have not tested this, but since IWN3* spreads into the degradative domain, does not colocalize well with SNX-1, and appears hyperactive, we think this mutant form of the protein may be SNX-1 independent and could continue to spread wider than wild-type RME-8 in a *snx-1* mutant.

The conclusion that Hsc70 uncoats SNX-1 is tenuous based on the existing data. A direct test of whether Hsc70 uncoats SNX-1 would be to examine SNX-1 accumulation in an Hsc70 mutant (or in Hsc70-binding RME-8 mutant). An alternate interpretation for the accumulation of SNX-1 in the absence of RME-8 could be that the large RME-8 protein obscures stronger SNX-1 binding sites or lipid interactions. If all binding sites are stably occupied, that would also lead to the reduced SNX-1 mobility by FRAP. The author’s show no direct evidence for turnover and need to phrase this as one interpretation among others.

This is a very good point; we now include alternate interpretations of our FRAP data, and phrase this differently in lines 345-347. In support of our idea that SNX-1 is a target, we did report in a previous study [10] that expression of a DNAJ domain active site mutant of RME-8, produced an effect on SNX-1 similar to the rme-8ts mutant. This does suggest that RME-8’s cochaperone activity is important for SNX-1 uncoating- a point I have added to the text 340-345

In general, it would be helpful if the AlphaFold models were discussed in the context of the individual figures, rather than saved for the discussion. For example, Fig. 8A-D would fit well in Fig. 1, while Fig. 8H-I could accompany the N-term deletion data of Fig 2. Also, how does the N-term deletion construct compare to the predicted AlphaFold structural elements like the three PH domains? The discussion hints that the deletion may have contained some of the predicted PH domains, but it is not made explicit with amino acid level comparisons.

We appreciate the reviewer’s enthusiasm for the alphafold results. ΔIWN1+2r is completely missing the two newly predicted PH domains, but also additional sequences. We prefer to keep the alphafold information to the discussion because the experiments were done before alphafold structural predictions were available. If we were starting now we would use different endpoints to better match predicted domains. To clarify our results have now used a more to-scale model and have highlighted pan-eukarya conserved domains as you suggested (Figure 1 and 2). Also, the structural model predicts three PH domains, two of which are outside of the previously defined lipid binding domain. We do not currently have experiments to test these additional PH domains cleanly, but they are completely removed. Hence we prefer to limit that idea in the discussion. We are excited to follow up on these results and will do more amino acid level comparisons but feel it is beyond the scope of the extensive data already in this paper.

Would the predicted lipid-binding surfaces of all three PH domains be able to interact with a flat or curved lipid bilayer at the same time, or do the authors think it more likely that they would interact in the context of an RME-8 oligomer, for example with each protein using a different PH domain to interact with the endosome membrane?

This is an excellent question and one we would like to pursue in future studies. The current structural model predicts that the second and third beta rich regions may have lipid binding domains that face each other, while the first one faces in another direction. Without experimentally derived structure data or any *in vivo* data we feel it is premature to speculate more about their potential orientations and ability to work simultaneouly.

Minor suggestions

Line 65-66 lacks a reference. Or is this a finding of this study? done

Line 70-72 lacks a reference. What and where were this previous evidence? done

In Figure 2G, there’s enough vertical space in the figure to spell out the full proposed function in two rows. Loc could be short for location or localization. Pos could be short for positive or positioning. I have altered figure 2

Line 155-157 & line 302 - This conclusion would depend on deleting the IWN domains individually, but this data does not appear in Fig. 3. Added data to figure 2, and see figure S3

Line 162 - More accurately RME-8 C-term, since the deletion removes other domains.

We changed the terminology as discussed above

Fig. 4A - The growth in the SNX-1 assay would be easier to see if the brightness was increased in this image (more similar to 4C)

I have adjusted the brightness.

Line 209-211 - Is this referring to Fig. 4C? Y2H is rarely described as a pulldown. It is referring to 4G. I have clarified the text lines 272-277
Line 213 - Is Figure. 6E supposed to refer to figure 9? 4I

Fig. 4D, the dark yellow N is hard to see in the dark blue background. Consider changing its color. Also, a similar sequence assembly for IWN3 would be helpful for interpreting the D1657K results.

good idea, I have altered the coloring in figure 4, adding an IWN3 alignment and a legend

Line 220-221, a conclusion re: SNX-1 interaction would also be helpful here.
done lines 244-245

Is the right side of Fig. 4G ever discussed?

Yes, in line 235 and 275

Lines 266-272 - The section on the in vivo IWN4* mutant results are hidden in the IWN3* section. It would be helpful to give these their own section with a separate heading.

We changed the header line 279. Organizationally we still would like to keep these results together.

Fig. 6A-D are mislabeled in the figure legend. Also, in A, 1 is equal distribution. 0 would be no interior intensity.

I have altered the figure legend. Also, for the graph, it is plotted on a log2 scale which is appropriate for ratiometric data. For example if something is 2x smaller or 2x larger the fold change is the same. If these results are plotted on a linear scale 2x smaller would be 0.5 and 2x larger would be 2. However on a log scale 2x smaller would be -1 and 2x larger would be 2. This way increases or decreases are treated the same

On a log2 scale 0= equal distribution -1= twofold less and 1= twofold more

2^0=1	2^1=2	2^-1=0.5

Fig. 6I - Are any changes significant? Also, the log2 scale is confusing if the authors want us to look for <1>.
see comment above, and thanks for the catch on missing P-values. I have added the p value stars back to the graph

Line 282 - on = of?

Yes, done

The data for RME-8(+) effect on RME-8(IWN3*) localization in Fig. 6E/K is not discussed in the text, although it appears in the conclusion in line 284.

It is discussed in lines 307-310

Line 288-290 - sentence fragments

Done now lines 313-315

The first paragraph of the discussion would be a strong intro paragraph.

Thank you, I put it in the intro, and reiterated some of it in the discussion

Paragraph 426-432 is not relevant to any of the data reported in this manuscript and would be more appropriate in an RME-8 review.
We prefer to keep this, as the C-terminal truncations of RME-8 would be presumed to be hyperactive, unless they could not bind substrate. Furthermore, we provide evidence that SNX-1 may be a target of RME-8 uncoating. Also, we feel it is important to remind readers that RME-8 is a co-chaperone and has no known enzymatic uncoating activity on its own- although I completely agree that an RME-8 review is in order!

Fig. 9B - Ready - Typo in occluded.
Thank you for pointing this out, we have fixed the spelling

No methods are given for structural prediction analyses.

We have added a paragraph for structural prediction analyses 574-579

Reviewer #2: Endocytosed cargo can be recycled by to the membrane or targeted to the lysosome for degradation. Previous work from Norris et al (2017) demonstrated that RME-8 and SNX-1 regulate endosome recycling, colocalize on endosome microdomains and restrict the localization of the ESCRT protein HGRS-1 to a degradative microdomain. The current study focuses on gaining a mechanistic understanding of RME-8 regulation and function. They identify and characterize IWN domains as important mediators of the RME-8 regulation and function. A phylogenetic analysis demonstrates that RME-8 is an ancient protein and identifies highly conserved regions that are suggestive of important roles in protein function. Particularly IWN domains, IWN1 and IWN2 in the N-terminal half of the protein between the N-terminal lipid binding domain and the centrally located DNAJ domain and IWN3 and IWN4 in the C-terminal half of the protein. Deletions of large portions of the protein containing IWN1/2 demonstrates that it is partially required for localization to endosomes. Deletion of the C-terminus containing the IWN3/4 domains results in stronger and more uniform RME-8 localization to endosomes indicating a role maintain localization to microdomains. Both deletions disrupt RME-8’s function in maintaining HGRS-1 degradative microdomains. The IWN3/4 region interacts with SNX-1 by yeast 2-hybrid, but inclusion of the DNAJ domain blocks SNX-1 binding under stringent conditions. An error prone PCR screen identified E1962K and N1966K mutations in the IWN4 domain that permit binding of SNX-1 in the presence of the DNAJ domain. The E1962K mutation also reduces the interaction of the IWN3/4 with the DNAJ domain indicating that an intramolecular interaction between IWN3/4 and DNAJ domains restricts SNX-1 binding. A similar PCR screen also identified a K1347E/K1356E mutation in the DNAJ binding that reduces the interaction with the IWN3/4 domains and permits stronger SNX-1 binding. Interestingly, a charge reversal mutation in the IWN3, D1657K, is more strongly activated regarding restricting HGRS-1 localization on endosomes, it diminishes the interaction with the DNAJ domain, however it does not permit SNX-1 binding in the presence of the DNAJ domain. The IWN3 D1657K mutation also results in a shift in RME-8 localization from being more peripheral to more interior in the coelomocytes. Colocalization with wild-type RME-8 demonstrates the change in localization, though wild-type RME-8 does partially rescue the localization defect consistent with potential dimerization. The IWN3 mutation also has reduced localization with SNX-1 but can rescue SNX-1 localization defects in a rme-8 mutant. SNX-1 shows increased localization to endosomes in a rme-8 mutant and FRAP shows reduced recovery in rme-8 versus wild-type suggesting a role for RME-8 in removal of SNX-1. Finally, using Alphafold structural predictions, they find that the IWN domains appear to be hinges connecting alpha solenoid structures providing insight into how the missense mutations in the IWN3 and IWN4 could disrupt function and demonstrating that the N-terminus appears to have three PH like domains that explains why the IWN1/2 deletion would affect membrane localization.

Strengths
This is a novel study that provides mechanistic insight into how a widely conserved regulator of endosome trafficking functions and provides many new avenues for further explanation. This study is nice in that it employs a variety of techniques to dig into RME-8 function using phylogenetic analyses, genetics, localization studies, binding assays, and structural modeling. The claims are backed by the data.

Weaknesses
The paper was a little difficult to read given errors in figure callout, misnumbering of figures and figures being out of order, though these are minor. It would be interesting to known how the point mutations affect cargo recycling, but this is not particularly necessary for this study.

Below are a list of typos and errors that need to be corrected.

P13 No figure callouts for Fig S3 (the real S3)

Lines 168, 171, 178, 218, and 328
S4 Figure is inserted between figures 4 and 5. Further, this is a supplement for figure 4, not figure 5.
Thank you for catching this, it has now been corrected
S5 Figure is also labelled as S4.
corrected
P14 line 198 Figure S3 callout should be for Figure S4
corrected
P14 line 213 Figure 6E callout appears to refer to Figure 4G?
corrected
P16 line 268 S4 Figure callout should be S5
corrected
P16 line 269 The triple Lysine CRISPR mutant does not match that in S5 Figure legend
Thank you for catching this, it has now been corrected
P16 line 270 The triple Lysine transgenic does match the residues listed in the Figure 5 legend.
Thank you for catching this, it has now been corrected- line 294-295
P21 line 369, odd phrasing “to our analyze”
corrected-
Figure 4C Label images as “assay” and “growth” as done in panels A and B
corrected
Figure 4D Only some of the color coding on amino acids in the alignment are described in the legend.
Thank you for catching this, I have simplified the coloring, added a legend, added an IWN3 sequence and updated the figure legend
Figure 4F Describe for the color coding for the sequence.
I have simplified the coloring to be consistent with 4C and added clarification to the figure legend
Figure 4H Define “V” and “wt” in the figure legend. Label images as “assay” and “growth”. The assay and growth images appear to be reversed as compared to the other Y2H panels.
I have added clarification to the figure legend and reversed the images in 4H to match the labels

Figure 4 legend, line 4 Should 1955 be 1962?
yes, thank you, I have corrected this error throughout the text
Figure 4 legend, line 5 R mutant appears to grow despite claim that it does not.
Yes, this was a mistake in the text. It does in fact grow as well as the lysine substitution

Figure 5 legend, IWN4* triple mutant, where did this come from and isn’t 1966 an Asp and not a Lys?
You are correct, 1966 is an Asp not a glutamic acid. The mutations were designed to further increase the introduced basic residues into the normally polar and/or acidic region.

Figure 5 legend Remove unnecessary P value indicators, there are no ** or **** in the figure. Same for other figure legends.
I prefer to have the range of p values listed even if they are not all used in a figure

Figure 6G and H, inset described in the legend is missing

I have altered the figure legend as I did not intend for there to be insets for these panels

Figure 6I, no statistics

I have added statistics, it was an error that they were not in the submitted draft

S3 Figure legend is incomplete

It was cut off, I have added it back

S5 Figure is labeled as a second S4

Thank you for catching this mistake, I have fixed this error

Reviewer #3: Summary

Cargos captured by early endosomes face two alternative fates. They are sorted either into the degradation pathway and deposited into the lysosomal lumen for degradation, or into the recycling pathways through recycling endosomes or Golgi. Previously, by studying early endosomes in the coelomocytes of the nematode C. elegans, these authors identified that the fate of the cargo is determined by whether it is at the proximity of one of the two adjacent and opposing microdomains established on the endosomal surface: the degradation microdomain coated by the ESCRT-0 complex, in which HRS/HGRS-1 is a major component, or the recycling microdomain coated by the RME-8/SNX-1 complex. RME-8 was proposed to further attract the Hsc70/DNAK protein (the uncoating protein) by the protein-protein interaction between the DNAJ domain of RME-8 and Hsc70. Each of these two microdomains occupies approximately half of the endosomal surface. They (Norris et al, 2017, PNAS) reported that the RME-8/SNX-1 complex acts to limit the spreading of the HRS microdomain. In snx-1(0) and rme-8(ts) mutants, increased endosomal coverage and intensity of HGRS-1–labeled microdomains, as well as increased total levels of HGRS-1 bound to membranes were observed. The HRS/HGRS-1 microdomain spreads to overlap with the recycling microdomain. On the other hand, in hgrs-1 loss-of-function mutants, the area covered by the RME-8/SNX-1 is not changed, indicating the active role of the recycling microdomain in establishing the microdomain pattern on endosomes.
In this new report, the authors have further investigated the mechanism of how RME-8 and SNX-1 establish the recycling microdomain and limit the spreading of the degradation microdomain. They analyzed the domain structure of RME-8 by alignment of the protein sequences of RME-8 family members in many species and by studying the AlphaFold prediction of the tertiary structure of RME-8. They further performed extensive structural-function analysis by employing three major approaches including (1) monitoring the endosomal localization pattern of the mutated or truncated proteins tagged with GFP-based reporters in various genetic backgrounds, (2) the yeast two-hybrid assay for protein-protein interaction, and (3) measuring the rescuing activity of the rme-8(b1023ts) mutant phenotype with multiple mutant forms of rme-8. These authors report that C-terminal to the DNAJ domain there are two IWN domains, IWN3 and 4. The region containing IWN3-IWN4 is able to bind to both the DNAJ domain of RME-8 and SNX-1. DNAJ and SNX-1 appear to compete with each other for the binding with IWN3-IWN4. In addition, mutations that change the charge of the amino acids in IWN3 and IWN4 both alter the binding affinity to DNAJ and SNX-1. Based on extensive mutational analyses, the authors propose a model of action. In this model, the IWN(3-4) region of one RME-8 molecule is proposed to interact with the DNAJ domain of a different RME-8 molecule, resulting in the formation of RME-8 oligomers, in which the DNAJ domain is hidden from interaction with Hsc70. By interacting with the IWN(3-4) domains, SNX-1 disrupts the inhibited conformation of RME-8, allowing the DNAJ domain to be exposed and accessible to Hsc70. The RME-8/Hsc70 complex then acts to uncoat the ESCRT-0 complex once it invades the RME-8 microdomain. Furthermore, this mode of action constitutes an activity cycle, since SNX-1 is also proposed to be a substrate of the RME-8/Hsc70 complex based on experimental evidence. The authors refer to this model as a “Ready Set Go model” for cyclical activation of RME-8.

I enjoyed reading this manuscript very much. How the fates of cargos are determined in the endocytic trafficking pathway was not well understood. This question is so universally important because if the regulatory mechanisms go awry, it would impact many developmental and health issues. The molecular mechanism proposed here is both original and of general interest. The model is well supported by experimental observations. The discovery will be welcomed by cell biologists, developmental biologists, and molecular biologists. I do not have any major concerns, yet there are a few specific issues that need to be further clarified. I would like to see my concerns addressed by either experiments (when specified) and/or additional explanations.

We are happy to see the manuscript received so well, thank you so much for these encouraging words

Specific concerns:

1. Lines 275-278, “Interestingly, the hyperactivity of RME-8(IWN3*) in reducing HGRS-1/Hrs levels on endosomes was blocked by endogenous wild-type RME-8, since we only observed this hyperactivity of RME-8(IWN3*) when endogenous RME-8 was removed by temperature shift of (b1023ts) mutants (Figure 5A” and B”, quantified in 5E)”.

This statement is given without showing the readers the proper control experiment, which is, what happens to the HGRS-1/Hrs levels in a wild-type strain expressing RME-8(IWN3*). This control is necessary.

We regret the oversight. We meant to include that data in the original submission. The data can now be found in supplementary figure 4

2. Regarding the microdomain localization of RME-8(IWN3*), Figure 6 (D and J) indicates that RME-8(IWN3*) expressed in the wild-type strain occupies part of the endosomal surfaces. However, Figure 6 (F and L) indicates that when RME-8(IWN3*) is co-expressed with RME-8, the endosomal localization of RME-8(IWN3*) is further expanded, yet that of RME-8 remains limited in its microdomain. How to explain the expansion of RME-8(IWN3*)? This expansion cannot be explained by the proposed inhibitory effect of RME-8 towards RME-8(IWN3*) (Lines 282-285), as the area that RME-8 is localized to does not change and is smaller than that of RME-8(IWN3*).

This is a good question. Given our model, we would propose that IWN3* is less clustered because it has reduced propensity to form oligomers with itself, and because it is more likely to interact with substrates in the degradative microdomain. The genetic results suggest that RME-8+ does have some ability to heterodimerize with IWN3* and inhibit its activity.

3. The discovery that “SNX-1 may be a substrate for RME-8/Hsc70 assembly/disassembly activities, in addition to acting as an activator of RME-8 uncoating activity toward the degradative microdomain.” is novel and exciting. It supports the model of a cyclic regulation of the microdomain occupation of the RME-8/SNX-1 proposed by the authors. To strengthen this model, I wonder whether the three IWN4 mutations that increase the binding affinity between RME-8 and SNX-1 in the yeast two-hybrid assay would affect the microdomain localization of SNX-1 in worms. In particular, in the Discussion, it is stated that “IWN4* improves the interaction with SNX-1 and is not hyperactive hinting that release of SNX-1 maybe an important part of the activation cycle of
417 RME-8”. Have these three mutations been tested for their in vivo effect on SNX-1 microdomain localization? If yes, what is the observation? If not, I would like to see them tested
This is an interesting question. We did not find any significant difference in colocalization of RME-8+/SNX-1 compared to IWN4*/SNX-1. Since RME-8+/SNX-1 colocalization is already nearly complete, we may not be able to detect any increase in colocalization between IWN4* and SNX-1. We have added this data to figure 6.

4. What is the mechanism(s) that restricts RME-8 on part of the endosomes, in its own microdomain? Do the authors have any speculations? The authors hint in the Discussion that uncoating of SNX-1 by the DNAJ/Hsc70 complex might act to restrict the RME-8 microdomain. However, in snx-1(0) mutants, RME-8 localization is peripheral rather than expanding the entire endosomes. Is there an explanation for this phenotype?

This is also an observation that we have been pondering, but we are still far from a full understanding. One contributing factor is likely RME-8 self-oligomerization acting to cluster RME-8 into a microdomain. The idea is that self-self interaction can create a microdomain island. In our recent review (Norris A, Grant BD: **Endosomal microdomains: Formation and function**. Current opinion in cell biology 2020, 65: 86-95.) we discuss this as a possible mechanism to aid in keeping functional microdomains separate and propose self-oligomerization as a likely contributing force also important for degradative microdomain formation, as ESCRT-0 component HRS is known to form high order homo-oligomers.

5. Also on the issue regarding the mechanism that restricts RME-8 to only part of the endosomes, RME-8(ΔIWN(3+4)) expands to the entire endosome in the wild-type strain. Does this expansion depend on the function of the endogenous wild-type RME-8? To answer this question, which will help distinguish whether the (ΔIWN(3+4) deletion causes a total loss of RME-8 function, could the authors characterize a rme-8(ΔIWN(3+4))::GFP homozygous mutant (CRISPR-generated) or the rme-8(b1023ts) mutants expressing rme-8(ΔIWN(3+4))::GFP for the endosomal localization pattern?

We have now added data relevant to this question to Figure S2. We had previously left this out because the results are hard to interpret because the endosomes collapse into irregular shapes. None of the truncations are fully functional. For instance see Fig S2 for quantification of coelomocyte size which we have found is a rough indicator of RME-8 function.

6. There are two phenotypes that need to be distinguished from each other. In the 2017 PNAS paper, it was reported that in rme-8(b1023ts) mutants, HRS expands its microdomain to most part of the surface of an endosome. In this manuscript, it is reported that in rme-8(b1023ts) mutants, there is an HRS overaccumulation phenotype. Is the overaccumulation phenotype the same as the expansion of the HRS microdomain? The illustration shown in Figure 3J seems to indicate that these are two different phenotypes. The overaccumulation phenotype is illustrated as that HRS signal intensity is increased inside its microdomain without the expansion of the microdomain. Is my understanding correct? Furthermore, the expression of RME-8(ΔIWN(3+4)) in the wild-type strain is reported to cause the overaccumulation of HRS on its microdomain, without any sign of HRS-microdomain expansion. What is the mechanism of this HRS overaccumulation, if HRS is still restricted in its usual microdomain?

Thanks for your question. In our previous work we showed two aspects of HRS response to loss of RME-8. We observed increased spreading of GFP-HRS around the endosome, and measured that even spread over a greater surface area, the fluorescence intensity of GFP-HRS increased, suggesting a higher percentage of HRS was assembled on the membrane than normal. We confirmed the overassembly interpretation using membrane fractionation, showing that in rme-8 mutants most HRS is assembled on the endosomes, and this is at the expense of the HRS pool free in the cytoplasm. Also, in our previous paper we measured HRS accumulation at two timepoints in the rme-8ts strain, one at 18hrs and one at 24-30 hours. With the 24-30 hours temperature shift of the rme-8ts mutant GFP-HRS is aggregated in clumps, but still shows increased intensity characteristic of overassembly. This overaccumulation of GFP-HRS at either timepoint is not restricted to its normal microdomain, as it mixes with the normally separate SNX-1 (Reported in the 2017 PNAS paper). We used the later timepoint for experiments in the paper under review because the phenotype is more extreme and easier to use in measuring differences. Also, in the new experiment to which you refer we used tagRFP-HRS, which unfortunately has more of a tendency to clump due to the red tag, which can interfere with measurements of spread. We needed to use a red label because all of the RME-8 truncation transgenes are green labeled. Given all of these technical issues we focused on the overassembly aspect of the phenotype here.

7. line 213, is Figure 6E the right figure to cite?

This has been corrected to 4I


8. Lines 264-266,“Conversely, in vivo we observe SNX-1 colocalization with RME-8 is dramatically lower for RME-8(IWN3*) than with wild-type RME-8 (Figure 6F-G”, quantified in 6J, illustrated in 6K)”.

--should the figure citation be (Figure 6G-H, quantified in 6K, illustrated in 6J)?

This has been corrected

9. Lines 268-270, “We tested CRISPR mediated endogenous alterations E1962K, and a triple lysine substitution at E1959, E1962, or N1966 (Figure S5)”.

-- Did not find Figure S5. Do you mean Figure S4?
-- There are two different figures that are labeled as Figure S4.

This has been corrected

**Have all data underlying the figures and results presented in the manuscript been provided?**
Large-scale datasets should be made available via a public repository as described in the *PLOS Genetics* [data availability policy](https://nam02.safelinks.protection.outlook.com/?url=http%3A%2F%2Fjournals.plos.org%2Fplosgenetics%2Fs%2Fdata-availability&data=05%7C01%7Cnorris%40dls.rutgers.edu%7C212a9948d12a4a4e981308da6b276587%7Cb92d2b234d35447093ff69aca6632ffe%7C1%7C0%7C637940114211404749%7CUnknown%7CTWFpbGZsb3d8eyJWIjoiMC4wLjAwMDAiLCJQIjoiV2luMzIiLCJBTiI6Ik1haWwiLCJXVCI6Mn0%3D%7C3000%7C%7C%7C&sdata=bbfPAtPwu7FETlZ5pIqsffQAMgxOacQpjkNThp6aSVM%3D&reserved=0), and numerical data that underlies graphs or summary statistics should be provided in spreadsheet form as supporting information.

Reviewer #1: None

Reviewer #2: Yes

Reviewer #3: Yes

PLOS authors have the option to publish the peer review history of their article ([what does this mean?](https://nam02.safelinks.protection.outlook.com/?url=https%3A%2F%2Fjournals.plos.org%2Fplosgenetics%2Fs%2Feditorial-and-peer-review-process%23loc-peer-review-history&data=05%7C01%7Cnorris%40dls.rutgers.edu%7C212a9948d12a4a4e981308da6b276587%7Cb92d2b234d35447093ff69aca6632ffe%7C1%7C0%7C637940114211404749%7CUnknown%7CTWFpbGZsb3d8eyJWIjoiMC4wLjAwMDAiLCJQIjoiV2luMzIiLCJBTiI6Ik1haWwiLCJXVCI6Mn0%3D%7C3000%7C%7C%7C&sdata=1CTO441QLPQbj%2BDbB8BQF14tMaxC%2FRxiQ%2BKcwC3eN8E%3D&reserved=0)). If published, this will include your full peer review and any attached files.


If you choose “no”, your identity will remain anonymous but your review may still be made public.


**Do you want your identity to be public for this peer review?** For information about this choice, including consent withdrawal, please see our [Privacy Policy](https://nam02.safelinks.protection.outlook.com/?url=https%3A%2F%2Fwww.plos.org%2Fprivacy-policy&data=05%7C01%7Cnorris%40dls.rutgers.edu%7C212a9948d12a4a4e981308da6b276587%7Cb92d2b234d35447093ff69aca6632ffe%7C1%7C0%7C637940114211404749%7CUnknown%7CTWFpbGZsb3d8eyJWIjoiMC4wLjAwMDAiLCJQIjoiV2luMzIiLCJBTiI6Ik1haWwiLCJXVCI6Mn0%3D%7C3000%7C%7C%7C&sdata=qavaOtBBOAhjiKO5bLW15zLTbIK1%2FfYJDNopILrQ55o%3D&reserved=0).

Reviewer #1: No

Reviewer #2: No

Reviewer #3: No
