# Supplementary material for: Mutagenesis and structural modeling implicate RME-8 IWN domains as conformational control points
Source: PLoS Genet. 2022 Oct 24;18(10):e1010296. doi: 10.1371/journal.pgen.1010296 (PMC9642905; doi:10.1371/journal.pgen.1010296)
Supplement: Attachment — Submitted filename: Reviewer response 0922.docx [file pgen.1010296.s025.docx]

Dear Reviewer

Thank you for your time and effort in making this manuscript better. Please see the point by point response and page numbers of edits (in bold) below.

Minor.

Fig 1B legend, indicate lipid binding domain (L), IWN domains (1-4) DNAJ (J). Obvious, but not always.

**done- line 617-618**

Fig S1B. Why are there numbers, lower case letters and spaces in the C. elegans RME-8 sequence? Seems like it would be more useful without these annotations from the multiple sequence alignment.

**done, I have entirely remade figure 2**

Fig 2A. Golgi are located in the cytoplasm as well as in the endosomes. Is this a stereotypical localization of Golgi in coelomocytes?

**Golgi are dispersed ministacks, they would not be inside, they would be throughout, I have altered cartoon to better reflect that**

Line 532 What is the meaning of the double zeta? **hydrophylic amino acids- I have added this info in parenthesis to make it more clear**

Line 620 Platted? Do you mean plotted? **yes!, corrected**

Fig 2. How is membrane to cytoplasm ratio measured? Do you draw a line across a vesicle membrane into the cytoplasm or draw a box in different regions of the coelomocyte? Do you measure one vesicle per animal? More details on how the differences are measured would be helpful for other doing similar experiments based on your work. Same with microdomain spread.

**I have added three paragraphs to the methods section addressing how I measured membrane versus cytoplasm, peripheral versus interior and microdomain spread- lines 578-599**

Since graphs are summaries of the data, an excel file with measurements for each of the graphs would increase transparency of the work.

**I have compiled the raw data into tab delimited text files and uploaded it as supplementary materials**

The callout for Fig S2 is after the callout for Fig S3 making it a little confusing (I thought you were missing the callout till I found it later in the text). **I want to keep S2 and S3 named as they are because they are addressing results from Figure 2 and figure 3 respectively**

Fig 3 and legend. Images A-C and D-F are labeled oppositely in the figure as they are described in the legend.

**I have fixed the legend- line 670-677**

Fig 3N the arrow is overlapping with the text

**done**

Fig 4B legend D1657 mutant is not described.

**done line 718-720**

Line 230. Should the Fig 4D callout be for 4C?

**yes, fixed line 230**

Fig 5F Text is overlapping with the drawing.

**fixed**

Line 295 Fig 5C callout should be for Fig S5C.

**the callout is correct for this one**

Fig 6D-F the inset of the higher magnification overlaps with D-F’’ which is visually unappealing. This should be physically separated or have a white outline that distinguishes it as separate from the underlying image.

**I respectfully disagree, I think the images are clear as is**

Line 318 What is the “n” in the Fig 6 callout?

**typo, it has been fixed -line 320**

Are there callouts for Fig 6 J-L?

**line 292**

Line 333-334 Fig 7 callouts are incorrect, should be A,B and H

**fixed, also fixed other figure 7 callouts in the same paragraph- line 336-340**
